# Research progress on assessment tools related to occupational fatigue in nurses: a traditional review

**DOI:** 10.3389/fpubh.2024.1508071

**Published:** 2024-12-06

**Authors:** Zhi Zeng, Sumei Zhou, Meng Liu

**Affiliations:** ^1^Department of Gastroenterology, Deyang People’s Hospital, Deyang, China; ^2^Department of Neurosurgery, Deyang People’s Hospital, Deyang, China; ^3^Pediatric Ward 2 (Children’s Blood/Cancer Ward), Sichuan Provincial People’s Hospital, Chengdu, China

**Keywords:** occupational fatigue, assessment tools, research progress, traditional review, nurse

## Abstract

Nurse occupational fatigue is a significant factor affecting nursing quality and medical safety. Scientific and effective assessment of occupational fatigue is beneficial for strengthening nurse occupational health management, improving the quality of life for nurses, and ensuring patient safety. This article provides a narrative review of the content, reliability, validity, characteristics, application status, and advantages and disadvantages of assessment tools related to nurse occupational fatigue. These tools include single-dimensional assessment scales (Fatigue Severity Scale, Chinese version of Li Fatigue Scale), multidimensional assessment scales (Fatigue Scale-14, Fatigue Assessment Scale, Multidimensional Fatigue Scale, etc.), and other assessment tools. Our review reveals limitations in existing occupational fatigue assessment tools, such as variability in accuracy and applicability across different populations, and potential biases. These findings underscore the critical role of these tools in nursing management and occupational health, advocating for continuous refinement and innovation. Future research should focus on developing more comprehensive, context-specific tools to address the multifaceted nature of nurse occupational fatigue. Nursing managers must carefully select appropriate tools to effectively identify and mitigate fatigue, thereby enhancing nurse well-being and patient care quality.

## 1 Introduction

In China, with the continuous development of the healthcare system and the increasing demand for health services, the nursing workforce has emerged as a pivotal force in the field of healthcare. According to statistics from the National Health Commission at the end of 2022, the number of registered nurses in China has exceeded 5.2 million, a figure that not only reflects the growing strength of the nursing workforce but also underscores the critical role nurses play in public health affairs (1, 2). However, the unique nature of nursing work has led to occupational fatigue becoming a widespread issue among the nursing population. Research indicates that over 80% of nurses experience varying degrees of fatigue within a week, a condition that entails not only physical exhaustion but also emotional and psychological strain (3, 4).

Occupational fatigue poses a profound impact on the physical and mental health, professional development, and patient safety of nurses. Prolonged fatigue often leaves nurses feeling physically and emotionally drained, manifesting as reduced work efficiency, emotional fluctuations, and deteriorating mental health, which may lead to burnout, diminished professional identity, increased turnover rates, and even adverse nursing incidents, posing a threat to patient safety (5–7). Therefore, selecting appropriate tools for evaluating occupational fatigue in nurses is of crucial practical significance for accurate assessment, early identification, and prevention.

Despite extensive research both domestically and internationally on the current status of occupational fatigue among nurses and its related factors, these studies predominantly focus on descriptive surveys and discussions of influencing factors, lacking a systematic exposition of commonly used evaluation tools. This paper aims to delve into commonly used occupational fatigue assessment tools both domestically and internationally, analyzing their application outcomes in clinical practice with actual cases. Our objective is to provide nursing managers with scientific and rational assessment means for more accurate evaluation of occupational fatigue in nurses. This will aid in the early identification of fatigue, providing a theoretical basis for targeted preventive and intervention measures. Consequently, it will enhance working environments and mental health of nurses, improve nursing quality, and ensure patient safety.

By systematically selecting and applying assessment tools, there is the potential to enhance the overall quality of the nursing workforce while reducing the negative impacts of occupational fatigue, thereby further promoting the sustainable development of the nursing profession. Therefore, the research in this paper will provide robust support for nursing management practices, contributing to the establishment of a healthier and more efficient nursing work environment.

## 2 The concept of occupational fatigue

Occupational Fatigue, first introduced by American psychologist Herbert Freudenberger in 1974, was designed to evaluate the work and mental health status of healthcare professionals (8). It is defined as a persistent state of physiological, emotional, and cognitive exhaustion and grief in the work environment, primarily caused by excessive workload and chronic stress. Since then, foreign scholars have further refined and enriched the connotation of occupational fatigue. Winwood and colleagues divided occupational fatigue into three dimensions: acute, chronic, and persistent (9). Additionally, the North American Nursing Diagnosis Association defines fatigue as a weariness caused by physical and mental labor, which results in decreased work and life capacities, and this fatigue cannot be alleviated by rest, highlighting its irreversible nature (10, 11). Drake and colleagues view occupational fatigue in nurses as a multidimensional state of physical and psychological burnout, triggered by insufficient recovery due to excessive demands at work (12).

Despite the above research, in China, there is currently no unified definition for occupational fatigue among nurses, indicating the need for further research and discussion to reach a consensus. Therefore, this study integrates relevant theories from occupational health psychology to provide a more comprehensive and in-depth theoretical framework for understanding occupational fatigue.

## 3 Theoretical framework

Occupational fatigue is a complex, multidimensional construct encompassing physical, emotional, and cognitive components (12). These dimensions collectively influence nursing performance and well-being, particularly in high-demand environments. To provide a robust theoretical foundation, this section integrates established frameworks and contextual factors.

### 3.1 Conservation of resources theory

Developed by Hobfoll, Conservation of Resources (COR) theory postulates that individuals are driven to acquire, maintain, and protect resources such as energy, time, and social support (13). In the nursing profession, the continuous depletion of these resources—often due to prolonged work hours, high patient acuity, and inadequate recovery opportunities—serves as a precursor to chronic fatigue and burnout. COR theory underscores the cascading nature of resource loss, wherein nurses lacking sufficient recovery mechanisms are disproportionately susceptible to fatigue-related consequences.

### 3.2 Effort-reward imbalance model

Proposed by Siegrist, the Effort-Reward Imbalance (ERI) model elucidates the relationship between occupational fatigue and imbalances in the effort-reward dynamic (14). This model posits that chronic exposure to high effort coupled with low reward erodes motivation, leading to emotional and physical exhaustion. For nurses, common examples include extended work shifts without sufficient recognition or financial compensation, further intensified in understaffed departments or high-pressure specialties such as emergency care. Empirical studies among Chinese nurses have identified ERI-related stressors as significant predictors of burnout and turnover intentions. The ERI model underscores the need for systemic organizational reforms to align effort with adequate rewards, not just in financial terms but also through professional development and peer recognition initiatives.

### 3.3 Cultural contextualization of fatigue

Cultural factors further shape the experience and expression of occupational fatigue. Studies in Asian nursing contexts reveal that hierarchical workplace structures and collectivist values amplify the pressures associated with occupational demands. For example, nurses in collectivist societies may prioritize team harmony over personal well-being, intensifying the depletion of emotional and cognitive resources. This cultural lens enriches our understanding of occupational fatigue by situating it within broader socio-cultural dynamics.

By integrating these theoretical constructs, the framework provides a comprehensive lens for analyzing occupational fatigue, encompassing individual, systemic, and cultural determinants.

## 4 Assessment tools for occupational fatigue

### 4.1 Uni-dimensional assessment tools

#### 4.1.1 Fatigue severity scale

The Fatigue Severity Scale (FSS) was developed by Krupp et al. in 1989 as a tool primarily used to assess the severity of fatigue experienced by individuals over the past week (15). It consists of 9 items, each rated on a 7-point scale ranging from 1 (strongly disagree) to 7 (strongly agree), with the total score being the average of these items. A score below 4 indicates no fatigue, a score between 4 and 4.9 suggests moderate fatigue, and a score of 5 or above denotes severe fatigue. The FSS has a Cronbach’s alpha coefficient of 0.89, indicating good internal consistency. In a study by Lee and Choi (16), the FSS Cronbach’s alpha coefficient for 234 Korean nurses was 0.91, further validating its reliability in this context.

##### 4.1.1.1 Reasons for selection

The FSS was selected due to its extensive application in measuring the severity of fatigue, aligning with the research objective of comprehensively reviewing tools related to occupational fatigue among nurses. Despite its limitations, the FSS provides preliminary insights into fatigue impacts in populations lacking standardized tools. Although limited in scope, it serves as a foundation for understanding the severity of fatigue before considering more comprehensive measurement methods.

The FSS is widely praised for its simplicity, ease of completion, and direct assessment of fatigue impacts. However, it has notable limitations, such as the inability to evaluate cognitive or social function impacts of fatigue, a narrow scope of applicability, and infrequent use among nursing populations. Notably, there is currently no validated Chinese version of the FSS, highlighting the necessity of considering cultural differences in future versions.

#### 4.1.2 Chinese version of the Lee fatigue scale-short form

The Chinese Version of the Lee Fatigue Scale-Short Form (C-LFS-SF), revised by Tsai et al. (17) in 2014, is a Chinese adaptation of the Lee Fatigue Scale (LFS) (18). This scale comprises 7 items, each scored on a continuum from 0 (no fatigue) to 10 (extreme fatigue), resulting in a total score range of 0 to 70, with higher scores indicating greater fatigue severity. The Cronbach’s α coefficient of the C-LFS-SF is reported to range from 0.97 to 0.99, showcasing excellent reliability, as evidenced in populations such as postpartum women (17), women with gynecologic cancers (19), and ICU nurses (20). Despite its robust reliability in these specific contexts, the C-LFS-SF’s application within the broader nursing population remains unvalidated, necessitating further verification of its reliability and generalizability.

##### 4.1.2.1 Rationale for selection

The choice of the C-LFS-SF in this review was driven by its relevance to the Chinese nursing context and its demonstrated reliability in assessing fatigue severity. As a culturally adapted tool, the C-LFS-SF offers a unique perspective on fatigue in Chinese healthcare settings, complementing other globally recognized measures. Its inclusion allows for a more nuanced understanding of fatigue among Chinese nurses, which is particularly important given the potential cultural and contextual differences in fatigue experiences.

### 4.2 Multi-dimensional assessment tools

#### 4.2.1 Fatigue scale-14

The Fatigue Scale-14 (FS-14), originally developed by British scholars Chalder et al. (21) in 1992, is a widely recognized instrument for assessing fatigue. It encompasses two primary dimensions: physical fatigue and mental fatigue, totaling 14 items. Items 1 to 8 are dedicated to evaluating physical fatigue, whereas items 9–14 focus on mental fatigue. The scale employs a 2-point scoring system, with “yes” scored as 1 and “no” as 0. Notably, items 10, 13, and 14 are reverse-scored, meaning “yes” is scored as 0 and “no” as 1. Consequently, the total score ranges from 0 to 14, with a score of ≥7 indicating the presence of fatigue. Higher scores suggest an elevated level of fatigue. The Cronbach’s α coefficient for the FS-14 varies from 0.88 to 0.90, with specific coefficients of 0.845 for the physical fatigue dimension and 0.821 for the mental fatigue dimension, signifying high internal consistency.

##### 4.2.1.1 Rationale for selection

The selection of the FS-14 in this review stems from its established reliability and widespread use in various professional groups, including healthcare workers. Its dual focus on both physical and mental fatigue makes it particularly suitable for assessing the multidimensional nature of fatigue among nurses. Additionally, the scale’s adaptability across different cultural contexts, as evidenced by its multiple translated versions, underscores its universal applicability (22–25). This tool’s sensitivity in capturing occupational fatigue and identifying fatigue symptoms among Chinese healthcare workers further justifies its inclusion in this review (4).

##### 4.2.1.2 Limitations and future directions

One notable limitation of the FS-14 is its inability to effectively differentiate between chronic fatigue and depression, which are often co-occurring conditions in healthcare workers. Future research could focus on refining the scale or developing complementary instruments to better distinguish between these overlapping symptoms. Additionally, ongoing studies should explore the generalizability of the FS-14 across diverse nursing populations and different work environments to ensure its continued relevance and applicability.

#### 4.2.2 Fatigue assessment instrument

The FAI, developed by American scholars Schwartz et al. (26) in 1993, is a widely recognized tool for assessing an individual’s physical and mental fatigue characteristics and levels over the past 2 weeks. The scale is comprised of four key factors: fatigue severity (11 items), fatigue environmental specificity (6 items), psychological consequences of fatigue (3 items), and the response of fatigue to rest and sleep (2 items), totaling 29 items. Each item is rated on a Likert 7-point scale, ranging from 1 (completely disagree) to 7 (completely agree), and the scores for each factor are expressed as x ± s, with a total score ranging from 4 to 28. Based on the average score for fatigue severity, fatigue is classified into four levels: less than 4 indicates no fatigue, 4 to 5 indicates mild fatigue, 5 to 6 indicates moderate fatigue, and 6 or above indicates severe fatigue. The Cronbach’s α coefficient for the FAI ranges from 0.70 to 0.92, demonstrating high reliability across various contexts.

Recent studies have shown that the FAI is extensively used in China to measure nurses’ occupational fatigue. For instance, Chen Haiyan et al. (27) examined occupational fatigue among nurses from five orthopedic hospitals in Nanchang, reporting a Cronbach’s α coefficient of 0.82. Similarly, two surveys on occupational fatigue among neurosurgical nurses yielded Cronbach’s α coefficients of 0.883 and 0.825, respectively, further validating the scale’s reliability (28, 29).

##### 4.2.2.1 Rationale for selection

The selection of the FAI was based on its robust theoretical foundation and extensive empirical validation in various clinical settings. While alternative tools do exist, the FAI’s multidimensional approach and the comprehensive coverage of both physical and psychological fatigue factors make it particularly suitable for assessing occupational fatigue among nurses.

##### 4.2.2.2 Limitations and future directions

Despite its reliability, the FAI’s complexity, characterized by multiple dimensions and items, poses challenges in terms of the time and effort required to complete the assessment, potentially affecting the quality and consistency of responses. To address this issue, future researchers are encouraged to simplify and revise the scale while preserving its core attributes and reliability.

#### 4.2.3 Multidimensional fatigue inventory

The Multidimensional fatigue inventory (MFI-20) is a self-assessment tool for occupational fatigue developed by Dutch scholars Smets et al. (30) in 1995 and validated in multiple populations (31–33). The scale comprises five dimensions: general fatigue, reduced activity, reduced motivation, mental fatigue, and physical fatigue. Each dimension has 4 items, totaling 20 items. The MFI-20 uses a Likert 5-point scoring system, where 1 to 5 represent “completely disagree,” “somewhat disagree,” “neither agree nor disagree,” “somewhat agree,” and “completely agree,” respectively. The total score ranges from 20 to 100, with items 1, 3, 4, 6, 7, 8, 11, 12, 15, and 20 being reverse-scored. A higher score indicates a more severe level of occupational fatigue. The Cronbach’s α coefficient of the MFI-20 is 0.84, and the AGFI is greater than 0.93, indicating high reliability. Ruishan et al. (34) revised the original scale to form the MFI-16, which was applied to measure the fatigue level of controllers, with a Cronbach’s α coefficient of 0.803. Miao et al. (35) translated and adapted it into the Chinese version of the MFI-20 in 2008, which was applied to military primary healthcare workers. The Cronbach’s α coefficient was 0.882, and the Cronbach’s α coefficients for the four dimensions of physical fatigue, mental fatigue, reduced motivation, and reduced activity were 0.867, 0.776, 0.476, and 0.687, respectively.

##### 4.2.3.1 Rationale for selection

The MFI-20 was selected as the assessment tool due to its wide applicability and validation in multiple populations. Compared to other existing fatigue assessment tools, the MFI-20 covers multiple dimensions of fatigue and demonstrates good reliability and validity across different cultural and geographic contexts. For instance, the MFI-20 has been proven effective and reliable in studies from the Netherlands, the United States, and China. Additionally, its simplicity and ease of administration make it a preferred tool in clinical and research settings.

##### 4.2.3.2 Limitations and future directions

Due to the variations in nursing research populations and geographical environments, the application of the MFI-20 to general nursing populations in China is limited. Additionally, current research primarily focuses on specific groups such as military personnel and controllers, necessitating further validation of its applicability to general nursing populations. Future research should expand the sample size, integrate different geographical and cultural contexts, and explore the major determinants of occupational fatigue, such as work organization, to validate the reliability and validity of the MFI-20.

#### 4.2.4 Occupation fatigue exhaustion recovery scale

The OFER was developed by foreign scholars Winwood et al. (9) in 2005 to measure individuals’ occupational fatigue levels. It consists of three subscales: chronic fatigue (5 items), acute fatigue (5 items), and recovery/persistent fatigue (5 items), totaling 15 items. Each item is scored using a Likert 7-point scale, ranging from “strongly disagree” to “strongly agree,” with scores from 0 to 6. Items 9, 10, 11, 13, and 15 are reverse-scored. The total score is calculated as the sum of the percentage scores of all items in the three subscales. Total scores in the ranges of 0–25, 26–50, 51–75, and 76–100 represent low, lower-medium, upper-medium, and high levels of fatigue, respectively. The Cronbach’s α coefficient of the scale is 0.85. The OFER has been widely used in assessing occupational fatigue among nursing populations in Korea (36), Japan (37), Lebanon (38), Saudi Arabia (39), and Italy (40). Mengyao et al. (41) applied the scale to measure occupational fatigue among Chinese nurses, and the results showed that the Cronbach’s α coefficient of the total scale was 0.91, while the Cronbach’s α coefficients of the subscales were 0.86, 0.91, and 0.75, indicating good internal consistency. Fang et al. (42) translated and validated the scale in a Chinese nursing population in 2018, with Cronbach’s α coefficients of 0.83, 0.85, and 0.86 for the three subscales, demonstrating high reliability.

##### 4.2.4.1 Rationale for selection

The Occupational Fatigue Efficiency Recovery (OFER) scale excels in distinguishing and measuring both acute and chronic fatigue, and it offers personalized recovery suggestions, making it highly effective for guiding the development and implementation of interventions aimed at alleviating nurses’ occupational fatigue. With a Cronbach’s α coefficient of 0.85, the scale demonstrates robust reliability and has been validated across multiple cultures, including Korea, Japan, Lebanon, Saudi Arabia, and Italy. This global applicability and reliability render the OFER an optimal tool for assessing occupational fatigue in nursing populations, providing crucial data to inform intervention strategies and enhance nurse well-being.

##### 4.2.4.2 Limitations and future directions

Despite its strengths, the OFER scale has several limitations. Primarily, its dimensions and items do not comprehensively evaluate occupational fatigue, omitting crucial factors such as work environment and personal lifestyle. This lack of comprehensive assessment limits the scale’s ability to provide a holistic view of fatigue, potentially affecting the accuracy and depth of insights gained. Future research should focus on expanding the scale to include additional dimensions that account for these missing variables. This enhancement would enable a more thorough evaluation of occupational fatigue, leading to more effective interventions tailored to the multifaceted nature of nurse fatigue. Additionally, the development of culturally adapted versions of the scale, particularly for emerging markets or underrepresented populations, could further improve its applicability and reliability across diverse nursing environments.

#### 4.2.5 Self-regulated fatigue scale

The SRF-S was developed by Nes et al. (43) in 2013 to evaluate individuals’ self-regulated fatigue levels. It comprises three dimensions: cognitive (5 items), emotional (6 items), and behavioral (5 items), totaling 16 items. The Likert 5-point scoring system is used, ranging from 1 (strongly disagree) to 5 (strongly agree). The total score ranges from 16 to 80, with higher scores indicating higher levels of self-regulated fatigue and ego depletion. The Cronbach’s α coefficient of the scale is 0.84. Cui et al. (44) validated the reliability and internal consistency of the scale among 353 Chinese nurses, with a Cronbach’s α coefficient of 0.842. Ligang et al. (45) translated and revised it into a Chinese version and validated it among Chinese youth, with a Cronbach’s α coefficient of 0.84 for the total scale and 0.68, 0.84, and 0.69 for the cognitive, emotional, and behavioral subscales, respectively. Jing et al. (46) applied the Chinese version to measure self-regulated fatigue among 451 nurses in a tertiary hospital in Jinan, with a Cronbach’s α coefficient of 0.846.

##### 4.2.5.1 Rationale for selection

The Self-Regulated Fatigue Scale (SRF-S) stands out due to its robust reliability, evident in its high Cronbach’s α coefficient of 0.84. This reliability has been consistently validated across different populations, including Chinese nurses and youth, demonstrating strong internal consistency (Cronbach’s α range: 0.84–0.846). The scale’s multidimensional approach, encompassing cognitive, emotional, and behavioral dimensions, provides a comprehensive assessment of self-regulated fatigue and ego depletion, making it particularly suitable for evaluating trait ego depletion levels among nursing populations. Its effectiveness in measuring self-regulation abilities and trait characteristics highlights its practical utility in fatigue management and intervention planning.

##### 4.2.5.2 Limitations and future directions

While the SRF-S effectively measures self-regulated fatigue, its focus on trait-like characteristics may overlook situational variations in fatigue regulation, necessitating future research to integrate dynamic and situational factors. Additionally, while the scale has shown strong reliability, further validation in diverse clinical settings and populations could enhance its applicability. Exploring cross-cultural validity and refining the scale’s predictive utility in relation to specific interventions could also improve its effectiveness in guiding tailored fatigue management strategies among nurses.

#### 4.2.6 Other scales

The “Self-Diagnosis Survey for Workers’ Fatigue Accumulation” was developed and released by the Japanese Ministry of Health, Labor, and Welfare in 2009. It is widely used to measure fatigue and excessive fatigue accumulation among occupational groups (47). It includes two dimensions: subjective symptom evaluation (13 items) and working condition evaluation (7 items), totaling 20 items. The Cronbach’s α coefficients for the scale and its dimensions are 0.892, 0.895, and 0.711, respectively. Tang et al. (48) conducted a survey on cumulative fatigue among 91,848 clinical nurses in China and found that the Cronbach’s α coefficients for the subjective symptom and working condition dimensions were 0.931 and 0.813, respectively, indicating good internal consistency. Although this scale has been widely used among manufacturing employees (49), its application in nursing populations is limited, and the detection rate is not high (50). Further multi-center large-sample empirical research is needed to validate its reliability. Additionally, the Fatigue Self-assessment Scale (FSAS) developed by Tianfang and Xiaolin (51) in 2007 is suitable for evaluating fatigue levels and types, as well as fatigue characteristics among healthy or sub-healthy populations. It includes two dimensions, with six main factors and 23 items. This scale is compatible with China’s local cultural background but is less commonly used abroad.

Both the “Self-Diagnosis Survey for Workers’ Fatigue Accumulation” and the “Fatigue Self-assessment Scale (FSAS)” offer robust reliability with high Cronbach’s α coefficients, suggesting strong internal consistency. These scales, particularly the former, have been validated in large-scale studies and provide comprehensive assessments of fatigue dimensions relevant to nursing populations. Their items and dimensions can inform the development and revision of nursing occupational fatigue assessment tools, providing valuable references for assessing occupational fatigue among nurses. Their items and dimensions can inform the development and revision of nursing occupational fatigue assessment tools, providing valuable references for assessing occupational fatigue among nurses. However, limited application in nursing and lower detection rates in some contexts highlight the need for further validation. Multi-center, large-sample studies are essential to enhance their reliability and applicability in nursing. Additionally, cross-cultural validation and refinement of predictive utility in relation to specific interventions are needed to improve effectiveness in guiding tailored fatigue management strategies.

### 4.3 Other occupational fatigue assessment tools

To evaluate the impact of cumulative work shifts on nurses’ occupational fatigue, Thompson (52) employed the psychomotor vigilance task (PVT) platform developed by Khitrov et al. (53). This experiment collected objective data on reaction time, vertical jump ability, and muscle group strength from nurses working three consecutive 12-h shifts to measure the degree and factors of occupational fatigue. Allik et al. (54) invented a wearable sensor that monitors heart rate and pulse to measure physical fatigue in real-time, providing impact data on cardiovascular parameters. Although this device has not been widely used, it offers a pathway for continuous real-time monitoring of physical fatigue. Furthermore, Aguirre et al. (55) applied machine learning algorithms to estimate fatigue levels (low, medium, and high) based on kinematic features and heart rate monitoring, achieving an accuracy rate of 82.5%. This method is particularly sensitive to predicting upper body fatigue. These tools primarily focus on objectively evaluating subjects’ physical fatigue levels, avoiding the subjectivity inherent in scale assessments and providing a more accurate reflection of fatigue states. It is anticipated that more scholars will validate these tools among nursing populations, leading to more scientific and objective methods for assessing occupational fatigue in nurses.

### 4.4 Comparative analysis of assessment tools

To critically evaluate the breadth and limitations of current assessment tools for occupational fatigue, a comparative analysis was conducted. Table 1 systematically presents key tools, detailing their dimensions, reliability, cultural adaptability, and limitations.

**Table 1:** Comparative analysis of nurse occupational fatigue assessment tools.

Tool	Dimensions evaluated	Reliability (Cronbach’s α)	Cultural adaptations	Limitations
Fatigue Severity Scale (FSS)	Physical fatigue	0.89–0.91	Predominantly validated in Western populations	Limited to physical fatigue; lacks assessment of emotional or cognitive fatigue
Chinese Version of the Lee Fatigue Scale (C-LFS-SF)	Fatigue severity	0.97–0.99	Validated in Chinese contexts	Limited generalizability to diverse populations
Fatigue Scale-14 (FS-14)	Physical fatigue, mental fatigue	0.845–0.90	Validated globally	Poor differentiation between chronic fatigue and depression symptoms
Fatigue Assessment Instrument (FAI)	Fatigue severity, environmental specificity, psychological consequences, recovery	0.70–0.92	Widely used in clinical settings	Complex structure and time-intensive nature limit its feasibility in high-paced environments
Multidimensional Fatigue Inventory (MFI-20)	Multidimensional (physical, mental, reduced activity, motivation, general fatigue)	0.84–0.87	Validated globally	Overlaps with depression symptoms; lacks situational focus
Occupation Fatigue Exhaustion Recovery Scale (OFER)	Acute, chronic, recovery	0.85–0.91	Cross-cultural validation in multiple countries	Lacks focus on external organizational factors influencing fatigue
Self-Regulated Fatigue Scale (SRF-S)	Cognitive, emotional, behavioral	0.84	Limited validation in nursing populations	Focuses on trait-based fatigue, potentially overlooking situational variability
Self-diagnosis survey for workers	Subjective symptoms, working conditions	0.89–0.93	Originally developed for Japanese contexts, later adapted for Chinese populations	Limited application in nursing; low detection sensitivity in certain occupational settings
Fatigue Self-Assessment Scale (FSAS)	Fatigue levels, fatigue types, fatigue characteristics	0.84	Tailored to Chinese cultural contexts	Limited international recognition; less commonly used outside China
Other tools	Real-time physiological metrics (e.g., heart rate, reaction time, muscle strength)	Not applicable	Emerging global interest	Requires further validation in nursing populations; reliant on technological infrastructure

### 4.5 Discussion on tool selection

#### 4.5.1 Strengths and weaknesses

Among the tools analyzed, the MFI-20 and OFER demonstrate high reliability and multidimensional insights, making them well-suited for comprehensive assessments. However, their limitations—such as the inability to capture environmental influences or overlap with mental health conditions—must be addressed in future studies. Similarly, while the C-LFS-SF offers strong cultural adaptability in Chinese contexts, it requires broader validation across diverse nursing populations to ensure its applicability.

#### 4.5.2 Future directions

There is an urgent need for culturally sensitive assessment tools that combine subjective self-report measures with objective metrics (e.g., wearable technology). Such tools can provide a more holistic understanding of fatigue dynamics in global nursing populations.

## 5 Discussion on main determinants of occupational fatigue among nurses and their integration into assessment tools

Nurses’ occupational fatigue is multifaceted, influenced by a range of factors including work organization, workload, social support, occupational stress, and individual health status. These determinants can vary significantly across different geographical and cultural contexts, necessitating a nuanced approach to fatigue assessment and management.

### 5.1 Cultural and regional variations

In Western countries, high workload and lack of social support often emerge as leading causes of occupational fatigue among nurses (56, 57). Conversely, in Asian countries, work organization and cultural norms may exert a more pronounced impact (58). For instance, the traditional emphasis on familial support and workplace hierarchies in Asian cultures can either mitigate or exacerbate fatigue (59), depending on how supportive these structures are perceived by nurses.

### 5.2 Work organization and geographical context

The assessment of occupational fatigue among nurses is intrinsically linked to work organization and geographical context. Variations in workload, shift patterns, and workplace support systems can significantly influence fatigue levels. Nurses working in high-stress environments, such as emergency departments or critical care units, often experience higher levels of fatigue compared to those in less demanding specialties (60, 61). Additionally, cultural and organizational factors, such as the availability of rest facilities and peer support networks, can play a crucial role in moderating fatigue levels (59, 62).

### 5.3 Broader determinants and implications for assessment tools

Despite the availability of various tools for assessing occupational fatigue among nurses, limitations persist. The development of occupational fatigue assessment tools in China started relatively late, primarily relying on the introduction of foreign tools and generic assessment scales, lacking tools specifically designed based on the local cultural context. Most existing occupational fatigue assessment scales have been developed by scholars from abroad, which may not align with the cultural background and regional environment in China. Thus, the reliability and validity of these tools within the domestic nursing population lack validation through large sample, multi-center studies.

Furthermore, there is currently no specialized assessment tool specifically developed for assessing occupational fatigue among nurses, indicating that the specificity and sensitivity of existing tools in measuring the extent of occupational fatigue need further analysis and validation. The broader determinants of occupational fatigue, such as workload, shift patterns, and organizational support, vary by region and significantly impact nursing fatigue. High patient-to-nurse ratios and resource constraints exacerbate fatigue, while supportive environments mitigate it. Future research should integrate these contextual factors into fatigue assessment tools for a more comprehensive view of occupational fatigue in nursing.

## 6 Prospects

This review presents an overview of commonly used occupational fatigue assessment tools for nurses domestically and internationally, revealing that these tools differ in terms of target populations, measurement dimensions, and evaluation focuses. Therefore, nursing managers should select appropriate assessment tools based on specific contexts. At present, there is a lack of specialized assessment tools for occupational fatigue in nursing professions, underscoring the need to develop instruments that incorporate local cultural characteristics to meet the unique needs of different nursing specialties (such as emergency care, psychiatry, ICU, etc.). Moreover, with the advancement of artificial intelligence technology, deep learning algorithms and various testing platform systems may be applied to the measurement of occupational fatigue among nurses. This would provide nursing managers with a more accurate, objective, and scientific description of the occupational fatigue status of nurses, assisting them in timely interventions to mitigate the detrimental effects of fatigue on their physical and mental health, thereby promoting the overall health and development of the nursing workforce.

## 7 Recommendations

To address the multifaceted nature of occupational fatigue, this section presents evidence-based strategies for enhancing both the assessment and management of fatigue within nursing. These recommendations are informed by the limitations identified in current tools and approaches.

1. Development of Comprehensive Fatigue Management Policies: National-level guidelines tailored to the unique demands of nursing specialties, such as critical care, endoscopy, and psychiatric nursing, should be established. These policies should incorporate regular fatigue assessments as part of occupational health programs, ensuring early detection and intervention.
2. Integration of Wearable Monitoring Technologies: Objective fatigue metrics, such as heart rate variability and pulse arrival time, captured through wearable devices, can complement traditional self-reported measures. These tools allow real-time monitoring, providing actionable insights for both nurses and managers to mitigate fatigue risks effectively.
3. Implementation of Evidence-Based Scheduling Models: Shift patterns should be redesigned to align with nurses’ circadian rhythms and recovery needs. For instance, limiting consecutive night shifts and ensuring adequate recovery intervals have been shown to significantly reduce fatigue levels among nurses.
4. Enhancement of Training and Support Mechanisms: Training programs must incorporate evidence-based approaches such as Mindfulness-Based Stress Reduction (MBSR), Cognitive Behavioral Therapy (CBT), and Energy Conservation Techniques (ECT) to help nurses mitigate occupational fatigue. These programs should include real-life case scenarios from high-stress nursing environments (e.g., ICU or emergency care) to ensure relevance and immediate applicability. Moreover, training can be supported by integrating digital platforms that provide on-demand stress management resources and fatigue monitoring. Peer support networks should be facilitated through regular reflective sessions moderated by trained professionals, providing a structured outlet for emotional and psychological support.
